# Occurrence of Virulence and Antibiotic Resistance in *Pseudomonas aeruginosa* Isolated from the Environmental Water from Tamaulipas, Mexico

**DOI:** 10.3390/antibiotics14121278

**Published:** 2025-12-17

**Authors:** Jessica I. Licea-Herrera, Abraham Guerrero, Paulina Guel, Virgilio Bocanegra-García, Gildardo Rivera, Ana Verónica Martínez-Vázquez

**Affiliations:** 1 Centro de Biotecnología Genómica, Instituto Politécnico Nacional, Reynosa C.P 88710, Tamaulipas, Mexico; 2 Centro de Investigación en Alimentación y Desarrollo (CIAD), Secretaria de Ciencia, Humanidades, Tecnología e Innovación (SECIHTI), Mazatlán C.P 82100, Sinaloa, Mexico

**Keywords:** *Pseudomonas aeruginosa*, irrigation water, rivers, virulence, antimicrobial resistance, Mexico

## Abstract

**Background/Objectives:** Antibiotic-resistant strains have been reported in aquatic ecosystems, with varying prevalence and resistance patterns by region. In Tamaulipas, Mexico, little information has been generated on this topic, making it difficult to estimate their potential risk to environmental and human health. Therefore, the objective of this study was to evaluate the presence and virulence of antibiotic-resistant strains of *Pseudomonas aeruginosa* in environmental water from Tamaulipas, Mexico. **Methods:** One hundred water samples were collected from different water bodies in Tamaulipas to identify *P. aeruginosa* by PCR and MALDI-TOF, virulence gene detection, antimicrobial susceptibility testing, and detection class 1 integrons. **Results:** In this study, 109 *P. aeruginosa* strains were isolated. Eight virulence genes were identified in 47.7% to 80.7% of the strains, with the *rhl*AB gene being the most frequent. The strains showed resistance or intermedia resistance to 10 of the 16 antibiotics tested, in a range of resistance values 0.9–66.2%. In total, 100% (109/109) were susceptible to ceftazidime (CAZ), gentamicin (GM), amikacin (AN), netilmicin (NET), tobramycin (NN) and norfloxacin (NOR), and 65.7% were resistant to ticarcillin/clavulanic acid and 53.5% to ticarcillin; the resistance to the remaining antibiotics was between 19.4% and 0.9%. The class 1 integron was not identified in any of the strains analyzed. **Conclusions:** *P. aeruginosa* in environmental waters of Tamaulipas showed potential to cause infections and low rates of resistance to most of the antibiotics tested. However, 20% were resistant to one of the most common treatments, which could pose a risk to public health.

## 1. Introduction

Freshwater is a widely used resource in domestic, agricultural, and industrial settings [1]. It is estimated that 70% of the water used is used to irrigate crops [2]; however, in recent years, the contamination of these tributaries with the presence of heavy metals [3,4], pharmaceuticals [5,6] and/or pesticides [7,8] has increased. Antibiotics have become a growing concern, as they have been reported in high concentrations in different aquatic environments worldwide [9,10]. Factors such as leaching of antibiotics from farms, agricultural runoff, or wastewater discharges into rivers increase the concentration of antibiotics in aquatic environments and, consequently, greater selective pressure on the bacterial communities present, contributing to the development of antibiotic-resistant bacteria (ARBs) [11,12]. Thus, the constant release (controlled or not) of antibiotic residues, even in small quantities, in aquatic and terrestrial ecosystems, induces resistance in bacteria and increases horizontal gene transfer [1,13,14,15].

Several studies have reported the presence of antibiotics and antibiotic-resistant bacteria in agricultural soil [16,17,18]. A recent study in agricultural soil in Tamaulipas, Mexico [19], reported the presence of *P. aeruginosa* strains with high percentages of virulence genes and antibiotic resistance. Although this can be attributed to several factors, water irrigation plays a crucial role in the distribution and accumulation of these antibiotic residues or resistant bacteria in soil [15,20]. In studies conducted in several countries, both antibiotic residues and resistant bacteria have been detected in rivers [6,20,21] and wastewater [18,21,22]. The prevalence of antibiotic-resistant bacteria and their resistance patterns vary across regions, so it is necessary to assess their presence and characteristics in each location.

Particularly in Mexico, in recent years, Solís-Soto et al. [23] published a study with river water samples in the state of Chihuahua in the northwest of the country, as well as the states of Veracruz and Puebla in the central zone. They identified the species *Enterococcus faecalis* and *E. coli*, which showed greater than 50% resistance to antibiotics such as ampicillin, vancomycin, and cefotaxime. This showed the presence of resistant bacteria in these rivers. For its part, Díaz-Zaragoza et al. [24] in the Ameca River in the center of the country reported *E. coli* strains with resistance percentages greater than 90% to antibiotics such as ampicillin, tetracycline, and streptomycin, widely used in veterinary medicine. In northeastern Mexico, Requena et al. [25] conducted a study of antimicrobial resistance in *E. coli* isolated from the Grande/Bravo River, which borders Mexico and the United States. Their results revealed that 85.7% of strains were resistant to at least one of the antibiotics tested, and 5.7% of strains were resistant to extended-spectrum β-lactamase (ESBL) producers. In these water samples from the Rio Grande/Bravo, the same research group identified other bacterial species resistant to one or more antibiotics. This generated interest in conducting more detailed studies on some of these species. One of them was *Pseudomonas aeruginosa*, which, given its importance in public health, represents a model organism of interest for evaluating its antibiotic resistance patterns, not only in the Rio Grande/Bravo but also in aquatic environments throughout the rest of the state of Tamaulipas.

The widespread occurrence of antibiotic-resistant bacteria in rivers may lead to the proliferation of antibiotic resistance genes (ARGs) that are considered new contaminants and pose potential health risks to humans worldwide [18,26]. Considering the above, this study aims to evaluate the presence of antibiotic-resistant strains and the virulence of *Pseudomonas aeruginosa* in environmental waters from Tamaulipas, Mexico.

## 2. Results

### 2.1. Sampling

Twenty-five municipalities in the state of Tamaulipas, Mexico, were visited to obtain a total of 100 environmental water samples (Table S1 in Supplementary Material). The water samples came 55% (55/100) of rivers, 16% (16/100) from irrigation canals, 9% (9/100) of lagoons, 8% (8/100) of streams, 5% (5/100) from marine environments, 3% (3/100) from dams, and 4% (4/100) from other bodies of water.

### 2.2. Pseudomonas aeruginosa Isolation

A total of 100 water samples were collected. From each sample, one to four colonies were randomly taken (depending on availability) in order to obtain a representative sample of the diversity of strains present in the samples, analyzing a total of 297 colonies. All strains were analyzed by PCR and MALDI-TOF to identify, confirming 49 water samples with *Pseudomonas aeruginosa* (49%, 49/100), of which a total of 109 (36.7%, 109/297) strains were obtained.

### 2.3. Detection of Virulence Genes

**Figure 1:**
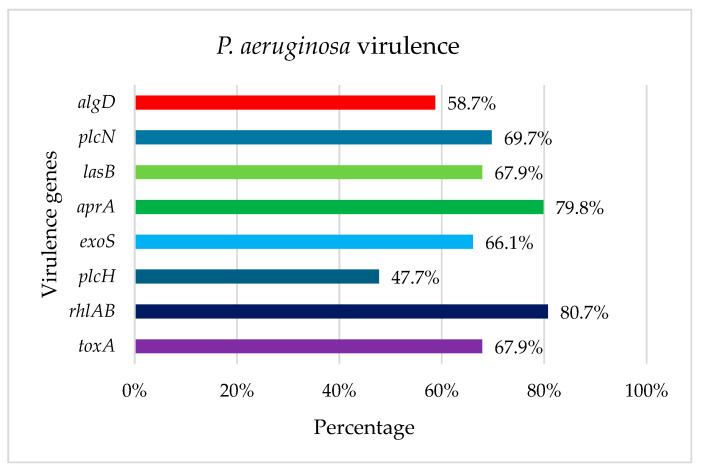
Prevalence of virulence genes in *P. aeruginosa* isolated from water environments from Tamaulipas, Mexico.

All eight virulence genes were detected in some of the *P. aeruginosa* strains. The *rhl*AB and *apr*A genes were more frequently detected, with 80.7% (88/109) and 79.8% (87/109), respectively. The *plc*H gene was the lowest present with 47.7% (52/109) prevalence (Figure 1).

The 36.6% (40/109) *P. aeruginosa* strains present all virulence genes included in this study.

### 2.4. Antimicrobial Susceptibility Testing

Overall, 23.8% (26/109) *P. aeruginosa* strains were resistant to one or more antibiotics tested, while 56.8% (62/109) strains showed intermediate resistance, and 19.2% (21/109) were susceptible to all antibiotics tested.

All strains (109/109) were susceptible to ceftazidime (CAZ), gentamicin (GM), amikacin (AN), netilmicin (NET), tobramycin (NN), and norfloxacin (NOR).

**Figure 2:**
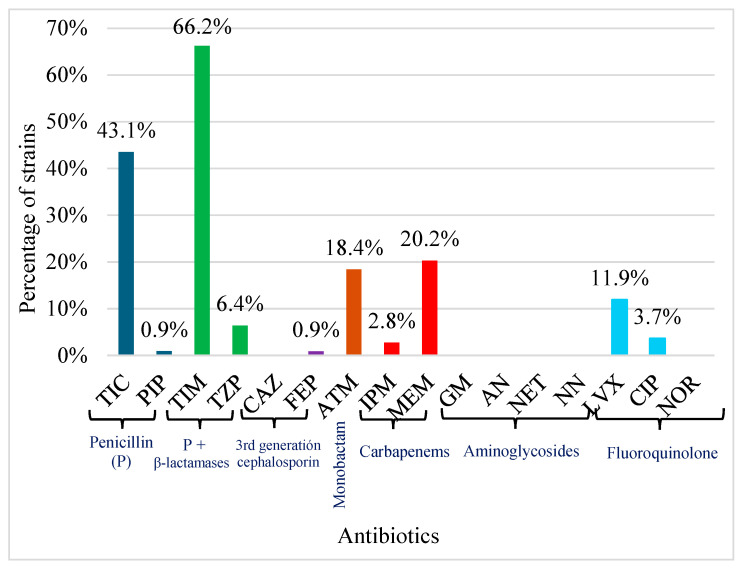
Percentage of *P. aeruginosa* strains resistant or intermediate resistant to the antibiotic panel. TIC: ticarcillin; PIP: piperacillin; TIM: ticarcillin with clavulanic acid; TZP: Piperacillin with tazobactam; CAZ: ceftazidime; FEP: cefepime; ATM: aztreonam; IPM: imipenem; MEM: meropenem; GM: gentamicin; AN: amikacin; NET: netilmicin; NN: tobramycin; LVX: levofloxacin; CIP: ciprofloxacin; NOR: norfloxacin.

The strains were resistance or intermediate resistance (R/I) to different antibiotics: (1) Regarding to Penicillin, 43.1% (47/109) were R/I to TIC and only 0.9% (1/109) to PIP; (2) combination of penicillin and β-lactamases inhibitor, 66.2% (72/109) were R/I to TIM, and 6.4% (7/109) to TZP; (3) about 3rd generation cephalosporin, only 0.9% (1/109) were R/I to FEP, the remaining strains were susceptible to this antibiotic family; (4) Concerning monobactam ATM 18.4% (20/109) were R/I; (5) in relation to carbapenems, 2.8% (3/109) to IPM and 20.2% (22/109) were R/I to MEM; (6) non or the strains were R/I to aminoglycosides family; (7) finally, related to fluoroquinolone family, 11.9% (13/109) were R/I to LVX and only 3.7% (4/109) to CIP. None of the strains were R/I to NOR (Figure 2 and Figure S1). No multidrug-resistant strains were detected (strains resistant to at least three different antibiotics from different classes).

Sixteen resistance patterns were found in the present *P. aeruginosa* strains. The resistance patterns with the higher number of strains were the combination of TIM + TIC (22 strains), followed by alone TIM (14 strains) and TIC (12) (Table 1).

**Figure 3:**
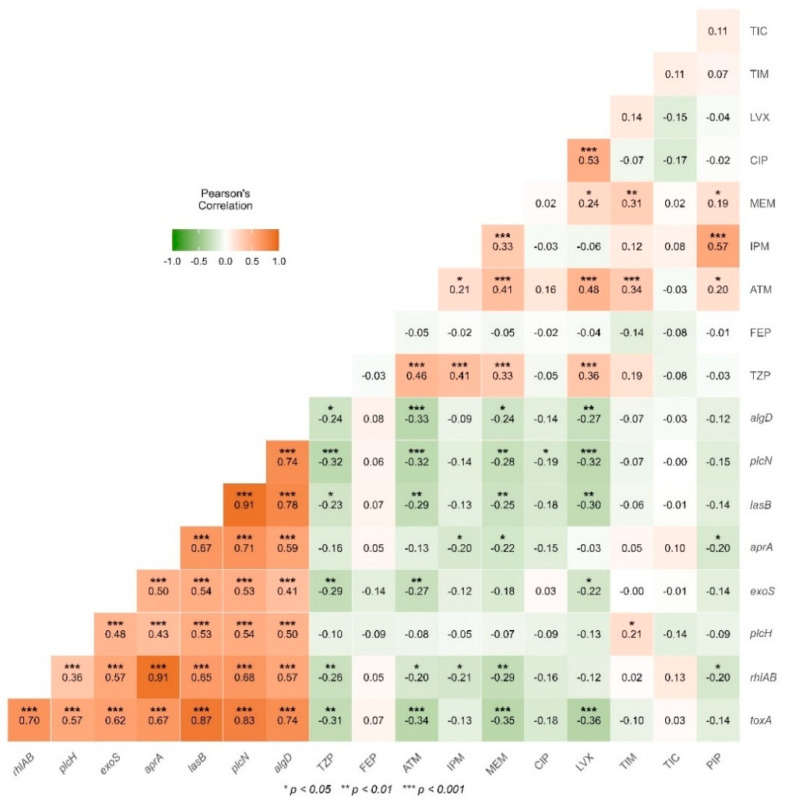
Pearson correlation between antibiotic resistance or intermediate resistance and virulence factor presence in *P. aeruginosa*. (Virulence factors: *alg*D, *exo*S, *plc*H, *plc*N, *tox*A, *apr*A, *las*B, and *rhl*AB; antibiotics: TIC: ticarcillin; PIP: piperacillin; TIM: ticarcillin with clavulanic acid; TZP: Piperacillin with tazobactam; FEP: cefepime; ATM: aztreonam; IPM: imipenem; MEM: meropenem; LVX: levofloxacin; and CIP: ciprofloxacin).

Pearson correlation analysis was carried out between antibiotic-resistant/virulence genes. The results revealed a strong positive correlation between all virulence genes (0.36 at 0.91 ***). As well as between IPM with PIP (0.57 ***) and IPM with MEM (0.33 ***), LVX with CIP (0.53 ***), LVX with ATM (0.48 ***), and LVX with TZP (0.36 ***). Also, TZP with ATM (0.46 ***), TZP with IPM (0.41 ***), and TZP with MEM (0.33 ***) (Figure 3).

On the other hand, a strong negative correlation was detected between *alg*D gene with aztreonam (ATM) (−0.33 ***), *plc*N gene with piperacillin with tazobactam (TZP) (−0.32 ***), *plc*N gene with aztreonam (ATM) (−0.32 ***), *plc*N gene with levofloxacin (LVX) (−0.32 ***). Also, *tox*A gene with aztreonam (ATM) (−0.34 ***), *tox*A gene with meropenem (MEM) (−0.35 ***) and *tox*A gene with levofloxacin (LVX) (−0.36 ***).

### 2.5. Detection Class 1 Integrons

The class 1 integron (*int*1) was not detected in any of the 109 *P. aeruginosa* strains analyzed.

## 3. Discussion

Several studies have been published reporting the presence of antibiotic-resistant bacteria in the environment; however, in many regions, no information has been generated on this subject. An example of this is the state of Tamaulipas, in northern Mexico, on the border with the United States, where, as far as our review went, little is known about resistant bacteria in the environment.

In a previous study by Requena et al. [27], water samples from the Bravo/Grande River on the border between Tamaulipas, Mexico, and the United States were analyzed. The results identified that 85.7% of *E. coli* strains were resistant to at least one antibiotic, and 5.7% were *E. coli*-producing extended-spectrum β-lactamase (ESBL), indicating that this body of water is a reservoir of antibiotic-resistant strains. Although this study focused only on the *E. coli* species, other bacterial species have been reported in the Bravo/Grande River [25] that could vary in their resistance patterns, so it would be important to evaluate them. Considering that the Bravo/Grande River is used to irrigate crops in this area, we hypothesize that irrigation water is a transmission route for the accumulation of resistant bacteria in agricultural soil. Therefore, our research group conducted a study in agricultural soil throughout the state of Tamaulipas, analyzing the antibiotic resistance profiles of *P. aeruginosa* [19]. As part of these published results, *P. aeruginosa* was present in 55% of the agricultural soil samples, identifying 40.8% of the strains in the central area of the state. Furthermore, the strains were resistant to only 3 of 16 antibiotics tested: 32.8% to ticarcillin, 40.8% to ticarcillin/clavulanic acid, and 2.4% to aztreonam. In parallel with this study, the current environmental water analyses were conducted, considering samples from water bodies near the agricultural soil samples. The current study has some sampling limitations; it would be advisable in future studies to carry out cross-sectional or longitudinal samplings to improve the representativeness of the samples.

The current study included 100 water samples from rivers, canals, streams, lagoons, and dams throughout the state of Tamaulipas, with a 49% prevalence of *P. aeruginosa*. Of the 109 strains identified, 53% were from the south of the state, mostly from the Guayalejo River.

Once the *P. aeruginosa* strains were identified, we first analyzed the presence of virulence factors that are involved with the pathogenicity potential (i.e., toxins, enzymes, and biofilm formation) that facilitate colonization and infection of the host cell [28,29]. The current study included eight of the most common virulence factors, identified in 47.7% to 80.7% of strains. If we compare the presence of virulence factors among strains isolated in Tamaulipas from water samples (47.7% to 80.7%) and soil samples (84.8% to 97.6%) [19], the prevalence is higher in soil. This could be because strains are exposed to different environmental conditions in water and soil, these environments being highly varied, with active substances and in varying concentrations, resulting in strains developing different resistance patterns.

In the current study, the gene with the highest prevalence was *rhl*AB with 80.7%, which coincides with other studies of *Pseudomonas* sp isolated from water or soil [19,30,31], being a gene that contributes to facilitating the invasion of bacteria into the host epithelial membrane, allowing access to other enzymes [32].

Another gene with high prevalence was *apr*A, with 79.8%, which degrades immune system proteins, facilitating systemic infection and tissue damage [33] (with a prevalence greater than 80% in other soil and water studies [19,30,31]).

An interesting finding is the contrast in the prevalence of the *plc*H gene, with 47.7% in samples of water and 93.6% in samples of agricultural soil from Tamaulipas [19]. This *plc*H gene, together with the *plc*N gene, encodes phospholipase C, a lytic enzyme responsible for modulating other virulence factors [32]. A similar case was observed with the *exo*S gene, whose prevalence varies widely, from 66.1% in water isolates to 90.4% in agricultural soil isolates from Tamaulipas. This *exo*S gene is associated with the strain’s invasion into mammalian cells and eventual death of the host cell by apoptosis [34], being the most prevalent gene among clinical isolates [35]. Interestingly, *P. aeruginosa* strains can be classified into two lineages, differentiated by the presence of either the *exo*U or *exo*S gene, which are usually mutually exclusive [36]. In the present study, the *exo*U gene was not searched for by PCR. Furthermore, strains with the *exo*U gene are associated with more severe disease due to their potent cytotoxic effects compared to strains with the *exo*S gene [36].

The *alg*D gene, which plays an important role in bacterial survival against the host immune response and drugs, was present in 58.7% of the strains isolated from water, which is a very low percentage compared to other studies, and *alg*D was present in more than 80% of the isolates obtained from water and soil [19,30].

In general, *P. aeruginosa* strains isolated from water presented a lower percentage of virulence genes compared to strains isolated from agricultural soil in nearby areas of the previous study by Licea et al. [19].

However, the picture changes when analyzing the resistance patterns of the strains. While *P. aeruginosa* strains from agricultural soil were only resistant to three of the 16 antibiotics tested, with a range of 4% to 32.8% [19], in contrast, the strains isolated from water in the current study showed some level of resistance to 10 of the 16 tested antibiotics, with a percentage ranging from 0.9% to 66.2%.

Strains isolated from water in this study were primarily resistant to TIM (66.2%) and TIC (43.5%), which coincides with the antibiotics most resistant to strains isolated from agricultural soil (TIM 40.8% and TIC 32.8%) in Tamaulipas as well. Most antibiotics used to treat *P. aeruginosa* infections must penetrate the cell membrane to reach intracellular targets [37]. For example, quinolone antibiotics, such as ciprofloxacin and levofloxacin, interfere with DNA replication by inhibiting DNA gyrase and topoisomerase IV [37,38]. Studies conducted in Brazil on *Pseudomonas* species isolated from soil and water reported 46 to 62% of strains resistant to CIP and LEV [30,39], while in the current study, less than 12% of strains were resistant (11.9% LEV and 3.7% CIP). Similarly, for IPM in Brazil, resistance was reported between 13 and 31% [30,39], while in the current study, only 2.8% of strains showed resistance.

Carbapenems such as imipenem (IPM) and meropenem (MEM) are routinely used to treat *P. aeruginosa* infections [40]. The current strains isolated from environmental water exhibited 20.3% resistance to MEM and 2.8% to IPM. We hypothesize that this resistance to MEM and IPM in environmental strains could be due to wastewater discharges from hospitals containing antibiotic residues and/or resistant strains. Observing the significant difference between the percentage of strains resistant to MEM (20.3%) and IPM (2.8%), we could hypothesize that MEM may be prescribed more frequently than IPM, resulting in higher levels of MEM residue in wastewater discharges and the environment. This could lead to strains developing a higher percentage of resistance to MEM. However, we lack specific information to support this. MEM in particular acts by easily penetrating bacterial cells and inhibiting the production of essential cell wall components, which causes cell death [41]. This resistance of strains isolated from water to meropenem (MEM) and imipenem (IPM) is relevant data for public health, since the World Health Organization (WHO) included carbapenem-resistant *P. aeruginosa* in the Priority 1 (critical) group of the list of priority pathogens for which research and development of new antibiotics is urgently needed [42,43]. On the other hand, it should be considered that while resistance to MEM and IPM monotherapies may decrease their effectiveness in *P. aeruginosa* infections, it has been found that their combination with antibiotics such as gentamicin can enhance their effectiveness [44].

When performing a correlation analysis between virulence genes and antibiotics to which the strains were resistant or intermedia resistance, some values of strong negative correlations were obtained (Figure 3). Negative correlation indicates that a pair of factors have an opposite action, where one increases and the other is reduced. In the current results of strains isolated from environmental water, only 4 of the 10 antibiotics to which they were resistant exhibited strong negative correlation values with any virulence gene.

In this study, the *tox*A gene was present in 67.9% of the strains and showed a strong negative correlation with the antibiotic aztreonam (−0.34 ***), levofloxacin (−0.36 ***), and meropenem (−0.35 ***). For its part, the *plc*N gene (69.7%) exhibited a strong negative correlation with aztreonam (−0.32 ***), piperacillin with tazobactam (−0.32 ***), and levofloxacin (−0.32 ***), while the *alg*D gene (58.7%) only had a strong negative correlation with aztreonam (−0.33 ***).

No positive correlation was found between virulence genes and antibiotics. However, a strong positive correlation was observed between all virulence genes (0.36 *** to 0.91 ***).

This study included a search for class 1 integron (*intl* 1), as it contains a genetic cassette that plays an important role in the transmission of antibiotic resistance genes, providing information on their potential transfer. However, class 1 integron was not detected in any of the strains analyzed, which coincides with the lack of MDR strains and the low percentage of antibiotic-resistant strains. This leads us to assume a low spread of gene cassettes that are resistant to various antibiotics. Therefore, it would be interesting to expand studies in this context.

The current study was the first to focus on *P. aeruginosa* in ambient water from Tamaulipas, Mexico, generating the first data on its antimicrobial resistance and virulence patterns. While it yields valuable information on its potential public health risk, it is important to expand this information with follow-up studies. In this regard, a complete genome analysis of these strains is planned for the near future, which will allow for the identification of all virulence and antimicrobial resistance genes present.

## 4. Materials and Methods

### 4.1. Sampling

**Figure 4:**
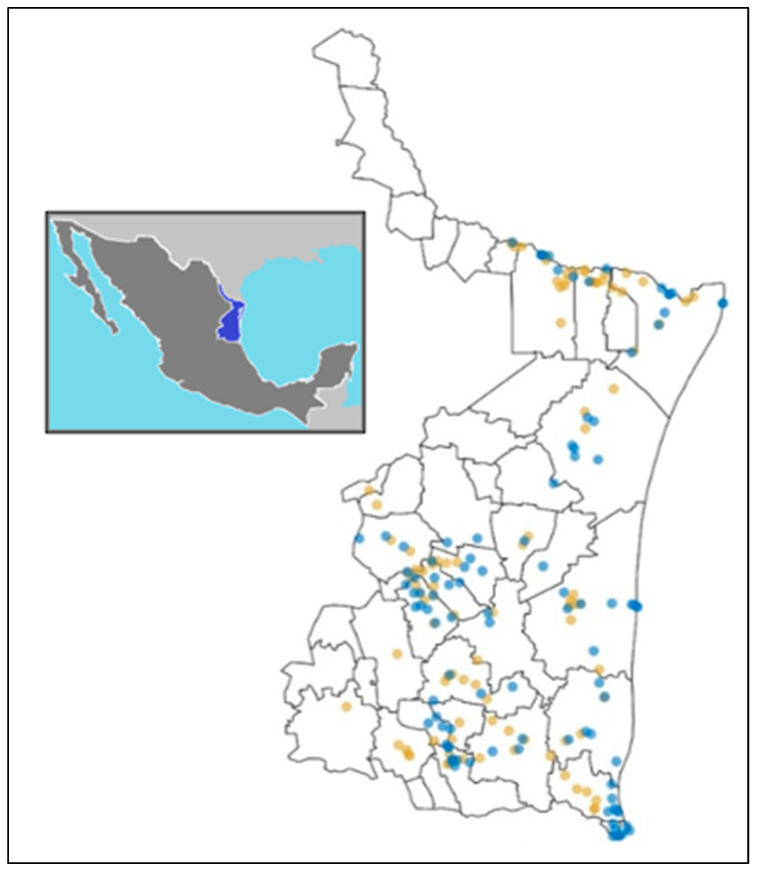
Environmental water sampling sites in the state of Tamaulipas. Blue: environmental water sample. Brown: agricultural soil sample in the study by Licea et al. [19].

Considering a parallel study by the same research group that analyzed agricultural soil samples (Figure 4) [19], environmental water sampling points related to these crop areas were selected. Irrigation canals, river water, and water dams were considered in this study. The water samples were collected from different municipalities in the state of Tamaulipas, Mexico, between the years 2021 to 2022. In each site, 1 L of water sample was taken at ~40 to 60 cm depth, collected with a sterile glass bottle. Each sample was labeled and stored in an icebox for transport to the laboratory in the Genomic Biotechnology Center-IPN.

### 4.2. Pseudomonas aeruginosa Isolation

Within 24 h of collection, each water sample was homogenized in peptone water (Becton Dickson & Co, Cuautitlán Izcalli, Mexico) at a 1:9 ratio and incubated aerobically at 37 °C for 24 h. They were then plated onto CHROMagar *Pseudomonas* (CHROMagar, Paris, France) plates and incubated at 37 °C for 24 h. Colonies with morphologically presumptive of *P. aeruginosa* were subcultured onto trypticase soy agar (TSA) (Becton Dickson & Co, Cuautitlán Izcalli, Mexico) plates for isolation and incubated 24 h at 37 °C.

Once the pure cultures were isolated and established, DNA extraction was performed by the lysis method [31]. DNA from individual isolates was used to amplify the *rpo*D gene for the *Pseudomonas* genus and the *ecf*X gene for the *aeruginosa* species using primers previously described by Güssow et al., and Talukder et al. [45,46]. The strains *P. aeruginosa* ATCC 27853^®^ and ATCC 9027^®^ were used as positive control.

The PCR reaction was carried in a volume of 25 µL, with 5× buffer (5X Colorless GoTaq^®^ Flexi Buffer, Promega, Madison, WI, USA), 25 mM MgCl_2_ (Magnesium Chloride Solution, 25 mM, Promega, Madison, WI, USA), 10 µM dNTPs (Bioline, Taunton, MA, USA), 10 µM primers, 5 U/µL Taq DNA polymerase (Promega, Madison, WI, USA) and sterile water. The amplification conditions were as follows: an initial denaturation at 95 °C for 15 min, followed by 30 cycles of 45 s at 95 °C, 45 s at 52 °C, and 45 s at 72 °C, with a final extension at 72 °C for 7 min. The amplicons were analyzed for 45 min under constant 100 V on 2.0% agarose gels with SYBR Gold (Invitrogen, Paisley, UK) and molecular marker (100 pb Promega, Madison, WI, USA).

The identification was confirmed by MALDI-TOF (Matrix-Assisted Laser Desorption/Ionization Time-of-Flight Mass Spectrometry), in a range of 2000 to 20,000 Da, and was generated with the Vitek MS Plus mass spectrometer (bioMerieux, Marcy l’Etoile, France). A confidence interval of 98–99% was considered acceptable for species-level identification (ID).

### 4.3. Detection of Virulence Genes

The DNA of *Pseudomonas aeruginosa* isolates were used as a template to amplify eight virulence-related genes (*alg*D, *exo*S, *plc*H, *plc*N, *tox*A, *apr*A, *las*B, and *rhl*AB) [34,35,36] (Table 2). A reaction mixture consisting of 5× buffer (5X Colorless GoTaq^®^ Flexi Buffer, Promega, Madison, WI, USA), 25 mM MgCl_2_ (Magnesium Chloride Solution, 25 mM, Promega, Madison, WI, USA), 10 µM dNTPs (Bioline, Taunton, MA, USA), 10 µM of each primer, 5 U/µL Taq DNA polymerase (Promega, Madison, WI, USA) and sterile water in a final reaction volume of 25 μL was used. PCR conditions were as follows: 95 °C for 3 min, 30 cycles of 95 °C for 1 min, 45 s at 55 °C, and 1 min at 72 °C were carried out, followed by a terminal elongation step at 72 °C for 5 min. The amplicons were separated electrophoretically through a 2% agarose gel in TBE 1x buffer and molecular marker (100 pb Promega, Madison, WI, USA).

### 4.4. Antimicrobial Susceptibility Testing

These procedures and evaluation methods were performed according to the Clinical and Laboratory Standards Institute (CLSI, Table S2) [49]. The isolates were homogenized in saline solution until the turbidity of 0.5 MacFarland standard. Cultures were swabbed on Mueller-Hinton agar (Becton Dickson & Co, Cuautitlán Izcalli, Mexico), and antibiotic disks were added. A panel of 16 antibiotics was tested: piperacillin-tazobactam (10/100 µg, TZP), piperacillin (10 µg, PIP) ticarcillin-clavulanic (75/10 µg, TIM), ticarcillin (10 µg, TIC), ceftazidime (30 µg, CAZ), cefepime (30 µg, FEP), aztreonam (30 µg, ATM), imipenem (10 µg, IPM), meropenem (10 µg, MEM), gentamicin (10 µg, GM), amikacin (30 µg, AN), netilmicin (30 µg, NET), Tobramycin (10 µg, NN) ciprofloxacin (5 µg, CIP), levofloxacin (5 µg, LEV), Norfloxacin (10 µg, NOR) (Sensi-Disk^TM^, Becton Dickson & Co., Franklin Lakes, NJ, USA).

After incubation at 37 °C for 18 h, the diameter of the zones of inhibition around each antimicrobial disk was measured in millimeters (mm), and the results were classified as resistant (R), intermediate (I), or susceptible (S) according to CLSI 2023 [49].

### 4.5. Detection of Class 1 Integrons

PCR was carried out using specific primers to detect the presence of intl1 (integron class 1) F: 5′-GGTCAAGGATCTGGATTTCG-3′/R: 5′-ACATGCGTGTAAATCATCGTC-3′ [50]. For the PCR mix prepared in a total volume of 25 µL: 5× buffer (5X Colorless GoTaq^®^ Flexi Buffer, Promega, Madison, WI, USA), 25 mM MgCl_2_ (Magnesium Chloride Solution, 25 mM, Promega, Madison, WI, USA), 10 µM dNTPs (Bioline, Taunton, MA, USA), 10 µM from each primer, 5 U/µL Taq DNA polymerase (Promega, Madison, WI, USA), DNA template and sterile water. The thermal cycling conditions were: initial denaturation at 95 °C for 1 min, 30 cycles of amplification (denaturation at 95 °C for 45 s, annealing at 54 °C for 45 s, and extension at 72 °C for 45 s), and a final extension at 72 °C for 7 min. The PCR products obtained were subjected to electrophoresis in a 2.0% agarose gel, added SYBR Gold (Invitrogen, Paisley, UK) at 100 V for 45 min. A molecular marker was run concurrently (100 pb Promega, Madison, WI, USA).

### 4.6. Statistical Analysis

A matrix was constructed using the data belonging to the virulence factors, which were evaluated in a dichotomic fashion, where 1 represented the presence of a gene and 0 represented its absence. As for the antimicrobials, resistance was represented with 1, the intermediate with 0.5, and susceptibility with 0. This matrix was used for the construction of the heatmap and the correlation matrix, both performed with R-Studio using R Version 3.4.1 (RStudio, Boston, MA, USA).

## 5. Conclusions

The *P. aeruginosa* strains isolated from environmental waters in Tamaulipas exhibited a high percentage of virulence genes, indicating their potential to cause infections. These strains showed low resistance rates to most of the antibiotics tested. However, 20% of the strains showed resistance to meropenem, one of the most common treatments for *Pseudomonas* infections, which could pose a risk to public health.

## Figures and Tables

**Table 1:** Resistant pattern found in *P. aeruginosa* strains isolated from the environmental water.

Patterns Number	Resistance Pattern	Number of Strains
1	CIP + LVX	2
2	TIC	12
3	TIC + ATM	3
4	TIC + MEM	8
5	TIC + MEM + ATM	4
6	TIM	14
7	TIM + MEM + ATM + CIP + LVX	1
8	TIM + MEM + ATM + LVX	5
9	TIM + MEM + FEP	1
10	TIM + MEM + IPM + TZP	1
11	TIM + TIC	22
12	TIM + TIC + LVX	1
13	TIM + TIC + MEM	3
14	TIM + TIC + MEM + ATM	5
15	TIM + TIC + PIP + ATM + MEM + IPM	1
16	TZP + ATM + IPM +MEM +TIM +TIC	1

**Table 2:** Primers of virulence-related genes used in the study.

Target Genes	Size (bp)	Primer Sequence 5′→3′	Reference
*alg*D	1310	ATGCGAATCAGCATCTTTGGT CTACCAGCAGATGCCCTCGGG	[47]
*exo*S	504	CTTGAAGGGACTCGACAAGG TTCAGGTCCGCGTAGTGAAT	[47]
*plc*H	307	GAAGCCATGGGCTACTTCAA AGAGTGACGAGGAGCGGTAG	[47]
*tox*A	352	GGTAACCAGCTCAGCCACAT TGATGTCCAGGTCATGCTTC	[47]
*apr*A	140	ACCCTGTCCTATTCGTTCC GATTGCAGCGACAACTTGG	[48]
*las*B	300	GGAATGAACGAAGCGTTCTC GGTCCAGTAGTAGCGGTTGG	[47]
*rhl*AB	151	TCATGGAATTGTCACAACCGC ATACGGCAAAATCATGGCAAC	[48]
*plc*N	466	GTTATCGCAACCAGCCCTAC AGGTCGAACACCTGGAACAC	[47]

## Data Availability

The original contributions presented in the study are included in the article; further inquiries can be directed to the corresponding authors.

## Supplementary Materials

The following supporting information can be downloaded at: https://www.mdpi.com/article/10.3390/antibiotics14121278/s1, Table S1. Water sampling sites in the state of Tamaulipas, Mexico. Table S2. CLSI breakpoints for *Pseudomonas aeruginosa*. Figure S1. Percentage of *P. aeruginosa* strains resistant or intermediate resistant to the antibiotic panel. TIC: ticarcillin; PIP: piperacillin; TIM: ticarcillin with clavulanic acid; TZP: Piperacillin with tazobactam; CAZ: ceftazidime; FEP: cefepime; ATM: aztreonam; IPM: imipenem; MEM: meropenem; GM: gentamicin; AN: amikacin; NET: netilmicin; NN: tobramycin; LVX: levofloxacin; CIP: ciprofloxacin; NOR: norfloxacin.
