# Supplementary material for: Occurrence of Virulence and Antibiotic Resistance in Pseudomonas aeruginosa Isolated from the Environmental Water from Tamaulipas, Mexico
Source: Antibiotics (Basel). 2025 Dec 17;14(12):1278. doi: 10.3390/antibiotics14121278 (PMC12729698; doi:10.3390/antibiotics14121278)
Supplement: Supplementary file 1 [file antibiotics-14-01278-s001.zip › antibiotics-3999903-supplementary.pdf]

supplementary material

Table S1. Water sampling sites in the state of Tamaulipas, Mexico

No.	Municipality	River	Coordinates		No.	Municipality	River	Coordinates	
			Latitude	Longitude				Latitude	Longitude
**1**	CD. Victoria	SLM	23° 46’ 58”	99° 11’ 58”	**51**	Gomez Farias	RG	22° 59' 16"	99° 08' 51"
**2**	CD. Victoria	SLM	23° 51’ 07”	99° 06’ 51”	**52**	Gomez Farias	RG	23° 01' 53"	99° 05' 35"
**3**	Güémez	SLM	23° 58’ 08”	99° 06’ 21”	**53**	Llera	RG	23° 08' 24"	99° 06' 50"
**4**	Padilla	SLM	24° 04’ 48”	99° 07’ 26”	**54**	Llera	RG	23° 18' 49"	99° 00' 15"
**5**	Padilla	SLM	24° 02’ 27”	98° 54’ 10”	**55**	G. Diaz Ordaz	RB	23° 13' 58"	98° 34' 40"
**6**	San Fernando	RSF	24° 47’ 24”	98° 09’ 21”	**56**	G. Diaz Ordaz	RB	26° 14' 09"	98° 34' 40"
**7**	Matamoros	RB	25° 29’ 31”	97° 46’ 16”	**57**	Reynosa	RB	26° 09' 10"	98° 23' 09"
**8**	Matamoros	RB	25° 40’ 45”	97° 35’ 21”	**58**	Reynosa	RB	26° 09' 03"	98° 22' 44"
**9**	Matamoros	RB	25° 53’ 34”	97° 29’ 47”	**59**	Reynosa	RB	26° 09' 03"	98° 22' 44"
**10**	Matamoros	RB	25° 53’ 10”	97° 31’ 08”	**60**	Hidalgo	SLM	24° 14' 02"	99° 36' 53"
**11**	Matamoros	PT	25° 53’ 04”	97° 31’ 08”	**61**	Hidalgo	SLM	24° 14' 59"	99° 26' 05"
**12**	Soto La Marina	SLM	23° 47’ 45”	97° 54’ 18”	**62**	Jiménez	SLM	24° 13' 04"	98° 29' 49"
**13**	Soto La Marina	SLM	23° 47’ 16”	97° 46’ 15”	**63**	Soto La Marina	SLM	23° 28' 28"	98° 01' 40"
**14**	Soto La Marina	SLM	23° 47’ 13”	97° 45’ 14”	**64**	Soto La Marina	SLM	23° 15' 32"	97° 59' 28"
**15**	Soto La Marina	SLM	23° 46’ 19”	97° 44’ 13”	**65**	Aldama	SLM	23° 09' 57"	97° 57' 14"
**16**	Soto La Marina	SLM	23° 46’ 19”	97° 43’ 51”	**66**	Aldama	SLM	22° 55' 51"	98° 04' 51"
**17**	Soto La Marina	SLM	23° 47’ 31”	98° 07’ 04”	**67**	Aldama	SLM	22° 54' 54"	98° 02' 49"
**18**	Soto La Marina	SLM	23° 45’ 36”	98° 12’ 18”	**68**	Aldama	SLM	22° 43' 48"	97° 52' 30"
**19**	Güémez	SLM	23° 55’ 17”	99° 00’ 27”	**69**	Altamira	SLM	22° 34' 33"	97° 54' 25"
**20**	Güémez	SLM	23° 56’ 14”	98° 56’ 10”	**70**	Altamira	S/O	22° 28' 48"	97° 54' 14"
**21**	Padilla	SLM	24° 00’ 53”	98° 46’ 54”	**71**	Altamira	S/O	22° 24' 46"	97° 53' 13"
**22**	Hidalgo	SLM	24° 10’ 44”	99° 18’ 54”	**72**	Altamira	S/O	22° 23' 56"	97° 52' 22"
**23**	Padilla	SLM	24° 05’ 53”	98° 51’ 46”	**73**	Altamira	S/O	22° 23' 13"	97° 51' 43"
**24**	San Carlos	SLM	24° 14’ 03”	98° 48’ 56”	**74**	Madero	GM	22° 17' 42"	97° 48' 21"
**25**	Reynosa	RB	26° 08’ 41”	98° 19’ 54”	**75**	Madero	GM	22° 15' 50"	97° 47' 09"
**26**	Casas	SLM	23° 43’ 45”	98° 44’ 36”	**76**	Madero	RG	22° 14' 24"	97° 49' 58"
**27**	CD. Victoria	SLM	23° 42’ 09”	98° 59’ 22”	**77**	Tampico	RG	22° 13' 37"	97° 51' 10"
**28**	CD. Victoria	SLM	23° 39’ 53”	99° 06’ 00”	**78**	Tampico	RG	22° 13' 35"	97° 52' 23"
**29**	CD. Victoria	SLM	23° 45’ 20”	99° 09’ 11”	**79**	Tampico	RG	22° 13' 33"	97° 53' 52"
**30**	CD. Victoria	SLM	23° 52’ 01”	99° 12’ 12”	**80**	Tampico	RG	22° 16' 22"	97° 53' 07"
**31**	Güémez	SLM	24° 00’ 30”	99° 17’ 27”	**81**	Tampico	RG	22° 18' 36"	97° 53' 49"
**32**	Soto La Marina	SLM	23° 52' 12"	98° 13' 45"	**82**	Altamira	RG	22° 23' 27"	97° 55' 51"
**33**	Villa de Casas	SLM	23° 40' 02"	98° 43' 59"	**83**	González	RG	22° 52' 58"	98° 30' 39"
**34**	CD. Victoria	SLM	23° 46' 18"	99° 14' 04"	**84**	Zaragoza	RG	23° 11' 13"	98° 47' 27"
**35**	CD. Victoria	SLM	23° 51' 57"	99° 14' 42"	**85**	Matamoros	RB	25° 49' 33"	97° 09' 05"
**36**	Güémez	SLM	23° 55' 20"	99° 17' 53"	**86**	Matamoros	RB	25° 49' 21"	97° 09' 20"
**37**	Güémez	SLM	23° 58' 07"	99° 14' 25"	**87**	San Fernando	RSF	24° 46' 01"	97° 59' 56"
**38**	San Carlos	SLM	24° 12' 28"	99° 01' 11"	**88**	San Fernando	RSF	24° 50' 31"	98° 09' 32"
**39**	Rio Bravo	RB	26° 03' 26"	97° 56' 27"	**89**	San Fernando	RSF	24° 51' 46"	98° 10' 26"
**40**	Rio Bravo	RB	25° 58' 18"	98° 03' 33"	**90**	San Fernando	RSF	25° 03' 07"	98° 04' 15"

(continuation) Table S1. Water sampling sites in the state of Tamaulipas, Mexico

**No.**	**Municipality**	**River**	**Coordinates**		**No.**	**Municipality**	**River**	**Coordinates**	
			**Latitude**	**Longitude**				**Latitude**	**Longitude**
**41**	Rio Bravo	RB	26° 00' 19"	98° 09' 58"	**91**	Aldama	RG	22° 52' 38"	98° 11' 53"
**42**	Reynosa	RB	26° 02' 52"	98° 16' 15"	**92**	Gonzalez	RG	22° 48' 48"	98° 32' 08"
**43**	Mante	RG	22° 44' 22"	98° 57' 13"	**93**	Gonzalez	RG	22° 47' 50"	98° 42' 47"
**44**	Mante	RG	22° 43' 44"	98° 59' 24"	**94**	Mante	RG	22° 43' 28"	98° 52' 06"
**45**	Mante	RG	22° 44' 45"	98° 59' 16"	**95**	Mante	RG	22° 41' 55"	98° 58' 07"
**46**	Mante	RG	22° 49' 16"	99° 00' 38"	**96**	Abasolo	SLM	24° 02' 47"	98° 22' 14"
**47**	Mante	RG	22° 49' 44"	99° 00' 31"	**97**	San Fernando	RSF	24° 36' 27"	98° 17' 53"
**48**	Mante	RG	22° 50' 33"	99° 01' 33"	**98**	Matamoros	RB	25° 45' 40"	97° 32' 50"
**49**	Xicoténcatl	RG	22° 56' 57"	98° 59' 52"	**99**	Matamoros	RB	25° 55' 40"	97° 34' 06"
**50**	Gomez Farias	RG	22° 58' 01"	99° 03' 08"	**100**	Río Bravo	RB	25° 01' 35"	98° 01' 39"

Table S2. CLSI breakpoints for *Pseudomonas aeruginosa*.

Antibiotic		Disk content	Interpretative categories and zone diameter breakpoints, nearest whole mm		
			S	I	R
Piperacilina-tazobactam	TZP	100/10 µg	≥21	15-20^	≤14
Ticarcillina-clavulánico	TIM	75/10 µg	≥24	16-23^	≤15
Ticarcillina	TIC	75 µg	≥24	16-23^	≤15
Piperacillina	PIP	100 µg	≥22	18-21^	≤17
Ceftazidime	CAZ	30 µg	≥18	15-17^	≤14
Cefepime	FEP	30 µg	≥18	15-17^	≤14
Aztreonam	ATM	30 µg	≥22	16-21^	≤15
Imiperem	IPM	10 µg	≥19	16-18^	≤15
Meropenem	MEM	10 µg	≥19	16-18^	≤15
Gentamicina	GM	10 µg	≥15	13-14^	≤12
Amikacina	AN	30 µg	≥17	15-16^	≤14
Netilmicina	NET	30 µg	≥15	13-14^	≤12
Tobramicina	NN	10 µg	≥19	13-18^	≤12
Ciprofloxacina	CIP	5 µg	≥25	19-24^	≤18
Levofloxacina	LVX	5 µg	≥22	15-21^	≤14
Norfloxacina	NOR	10 µg	≥17	13-16^	≤12

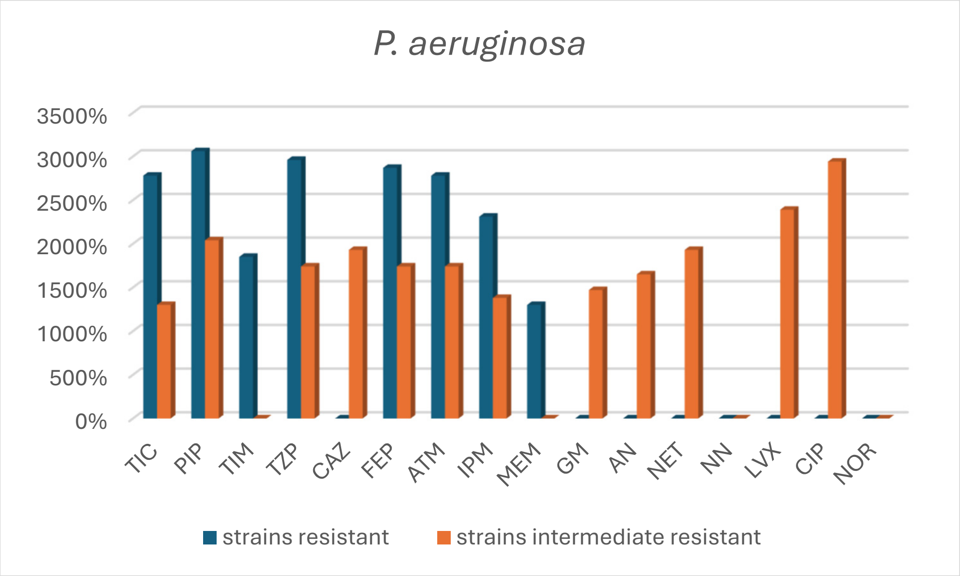

Figure S1. Percentage of *P. aeruginosa* strains resistant or intermediate resistant to the antibiotic panel. TIC: ticarcillin; PIP: piperacillin; TIM: ticarcillin with clavulanic acid; TZP: Piperacillin with tazobactam; CAZ: ceftazidime; FEP: cefepime; ATM: aztreonam; IPM: imipenem; MEM: meropenem; GM: gentamicin; AN: amikacin; NET: netilmicin; NN: tobramycin; LVX: levofloxacin; CIP: ciprofloxacin; NOR: norfloxacin.
